# Beyond privacy vs. health: a justification analysis of the contact-tracing apps debate in the Netherlands

**DOI:** 10.1007/s10676-020-09555-x

**Published:** 2020-10-10

**Authors:** Lotje Elizabeth Siffels

**Affiliations:** Faculteit Filosofie, Theologie en Religiestudies, Radboud Universiteit, Erasmusplein 1, Postbus 9103, 6500 HD Nijmegen, The Netherlands

**Keywords:** Corona-app, Digital health, Justification framework, COVID-19, Moral repertoires, Common good, Public values

## Abstract

In the Netherlands, as in many other nations, the government has proposed the use of a contact-tracing app as a means of helping to contain the spread of the corona virus. The discussion about the use of such an app has mostly been framed in terms of a tradeoff between privacy and public health. This research statement presents an analysis of the Dutch public debate on Corona-apps by using the framework of Orders of Worth by Boltanski and Thévenot (1991). It argues that this framework can help us to move beyond the dichotomy of privacy vs. public health by recognizing a plurality of conceptions of the common good in the debate about contact-tracing apps. This statement presents six orders of worth present in the Dutch debate: *civic, domestic, vitality, market, industrial* and *project,* and argues that the identification of which common goods are at stake will contribute to discussions about the use of this technology from a standpoint with a richer ethical perspective.

## Roadmapping beyond privacy: two approaches for mapping ethical considerations involving contact tracing apps

As many countries across the globe are struggling with Covid-19, a discussion is taking place about the possible use of (a wide variety of) contact tracing apps. The goal is to gain insight in the spread of Covid-19 which, in many cases, requires location data and biometric information. Most concerns about these apps in the discussion focus on privacy as an individual right to control over one’s information.^1^ However, we believe that this discussion should be broadened to include other ethical considerations and a richer understanding of privacy as a public value.

We present two research statements that contribute to the discussion by offering considerations ‘beyond privacy’ when evaluating the development and implementation of contact tracing apps. The first, *‘Contact tracing apps: an ethical roadmap’,* presents a roadmap for the ethical evaluation of contact-tracing apps. It raises three ethical concerns—privacy, Big Tech dependency and coercion—by exploring three scenarios (Lanzing 2020, this issue). The second, ‘*Beyond Privacy vs. Health: a justification analysis of the contact-tracing apps debate in the Netherlands*’, shows how a justification analysis of the debate about contact tracing apps, using the framework developed by Luc Boltanski and Laurent Thevenot, can enable us to recognize a plurality of common goods at stake (Siffels 2020, this issue).

Both statements are part of the ‘Digital Good’ project, an interdisciplinary research project that focuses on the disruption of health as we move into the digital era. The project investigates ways of approaching the digitalization of health from a standpoint of the common good, rather than one of individual privacy. Its aim is to look for governance frameworks that foreground collective welfare and public values, while acknowledging a plurality of conceptions of the common good at work in the digitalization of health.

## Beyond privacy vs. health: a justification analysis of the contact-tracing apps debate in the Netherlands

### Lotje Siffels

**Privacy vs. health**

Governments and health authorities across the globe have proposed the use of contact-tracing apps as a means of helping to contain the spread of the corona virus and easing lockdown measures.^2^ Among scholars, experts and NGOs, there is ongoing discussion about the ethical aspects of the use of such apps. In the Netherlands, as in many other nations, this discussion has mostly been framed in terms of a tradeoff between privacy and public health.^3^ This dichotomous framing can be harmful in several ways. First, it implies that when privacy will have been ‘taken care’ of, the task of ethical reflection for this technology will be complete. This concern goes hand in hand with the fact that privacy is conceptualized as an individual good which can be protected with anonymization. Therefore it seems that if the app correctly anonymizes the data, everything is well. This frame crowds out other ethical considerations. Second, it frames a dilemma of two values as a zero-sum game. It implies that regard for one of the two means a disregard for the other. Often in this trade-off, health wins. Advocates for the Corona-app may argue: ‘corona requires a temporary sacrifice of privacy’.^4^ By the same token, critics of the corona app often also used this frame when they argued that health is good, but not if it costs us our right to privacy.^5^ Third, this dichotomy does not only frame privacy as opposite to health, but also frames private concerns as opposite to public concerns. The frame therefore misses out on ways in which the interest of private individuals can align with public interests, and misses out on ways in which both health and other concerns could be protected.^6^

## Orders of worth

To move beyond a dichotomy of private and public, privacy and health, the framework developed by the French sociologists Luc Boltanski and Laurent Thévenot is very useful. Boltanski and Thévenot argue in their book *On Justification* (2006) that there is a plurality of ways people conceptualize the common good when they justify their actions. This set of common goods is not individual, but shared in a cultural context. Which common good one mobilizes depends on the situation. In different situations people have different dispositions and will refer to different kinds of common good. Boltanski and Thévenot discuss these common goods as ‘orders of worth’. Each order contains a hierarchy of worth, where something or someone’s worth is measured in terms of relative distance from others in that order, and relative to the common good of the order in question. Boltanski and Thévenot describe six orders of worth in western liberal society: ‘inspired’ (where the common good is inspiration and a sense of the sublime), ‘domestic’ (respecting tradition and hierarchy), ‘fame’ (gaining public acclaim), ‘civic’ (acting for the good of society), ‘market’ (competition and economic growth) and ‘industrial’ (efficiency and expertise).^7^ This list of orders is not exhaustive. In recent years, new conceptions of common good have been identified by scholars using this framework including ‘project’ (innovation), ‘green’ (environmentalism) and ‘vitality’ (health) (Boltanski and Chiapello 2005; Lafaye and Thevenot 2017; Sharon 2018). Each order has its own conception of the common good, and in each order certain values are seen as more worthy than others. For example, in the civic order, the common good is conceptualized as acting for the good of society, and the values of solidarity, equality and inclusivity are foregrounded. In the industrial order, the common good is conceptualized as increased efficiency, and values like expertise, optimization and functionality are commonly used.

By analyzing how people justify their actions and decisions, we can recognize which conceptualization of the common good they are using, i.e. which order of worth they mobilize. This helps us to map which kinds of common good are at stake in this debate. Such a map provides a plurality of common goods, but is limited at the same time. There aren’t endless possibilities of orders of worth. Every order has to be identified by doing empirical research into a debate. Informed by what one encounters in the empirical investigation, all justifications should have its place on this map. The map is not meant to be exhaustive, however. Different orders of worth will be used in different places, historical moments and contexts. New orders can emerge. If there are instances where another conception of common good is used that had no place on the map of orders of worth yet, this could be added as a new order of worth (Boltanski and Thévenot 2006). The ‘green’ order is an example of this, having been added years after the initial publication of the framework (Boltanski and Thévenot 2017). By the same token, older orders can become obsolete if they are no longer mobilized. Because of its recognition of a plurality of ethical considerations, this framework allows us to analyze the debate with a richer framework at hand. It is a useful tool for going beyond the dichotomy of privacy vs. public health. First of all because it does not distinguish first and foremost between private vs. public interests. All justifications are based on a conceptualization of the common good that is commonly recognized by a group of people. Secondly, the justification framework is useful because it can identify more kinds of common good than just privacy and health, that can exist in the same deliberative space (Sharon 2018) (Table 1).

**Table 1:** Orders of worth present in the corona-app debate in the Netherlands

Order	Common good	Example
Civic	Collective well-being	‘This can only be done when we handle privacy carefully. It can only be done with trust from society’^a^
Domestic	Tradition and hierarchy	‘That is how we do things here’
Vitality	Greater health	‘Better detection of infections can help prevent a new revival of the outbreak’
Market	Economic growth	‘[we need to] get to a situation in which we try to make the economy function as well as possible’
Industrial	Increased efficiency an expertise	‘Apps may be able to largely automate the source and contact research(..), you can trace contacts where we may not be aware of or have forgotten, thereby speeding up the process
Project	Innovation	‘It's 2020! We are in the twenty-first century and I think (…) that some of the ways we work now are still very deep, deep from the last century.’^b^

^a^Original dutch: ‘dat kan alleen als we heel zorgvuldig omgaan met privacy. Het kan alleen met vertrouwen van de samenleving’

^b^M. Heidenrijk at Pakhuis de Zwijger on 08/05/2020: https://www.youtube.com/watch?v=gFo_oS3wlR4&t=2240s (accessed 04–06-2020). Original Dutch: ‘ja het is gewoon 2020! We zitten gewoon in de 21^ste^ eeuw en ik vind, dat dat wel een stukje kritiek, dat sommige manieren hoe wij nu werken nog heel erg diep, diep vanuit de vorige eeuw zijn.’

In the debate on corona-apps, the appeal to privacy tends to be a mobilization of the ‘civic’ order, where it is valued to place the common good above individual interests, and where equality and solidarity are valued. When privacy is defended as a civil right, the civic order is mobilized. The appeal to public health and safety tends to be a mobilization of the ‘domestic’ order, which is mobilized by emphasizing the state’s responsibility to protect its citizens. However, in the Dutch debate the argument for health is sometimes an appeal to the ‘vitality’ rather than the ‘domestic’ order. The description of an ‘intelligent lockdown’, where each citizen is asked to take individual responsibility for behaving in a way that improves the overall goal: public health, appeals to vitality. The necessity of the corona-app is justified by appealing to vitality, for example when the Dutch minister for health says: ‘Better detection of infections can help prevent a new revival of the outbreak’.^8^ However, the main argument is that the app is a necessity to lift the policy measures that are implemented to stop the spread of corona. The app then, is presented as offering a compromise between the order of vitality and other orders that were traded off for the good of vitality during the lockdown, most prominently the market order. Simply put, it is argued that if we want both health and economic growth, we need the app.^9^

Next to vitality and market, another order that is often used to justify the use of an app in this debate is the order of industry, where increased efficiency is valued. The necessity of the app in the Dutch context is presented as the need to automate contact-tracing done by the Dutch local health services (GGD), emphasizing that this can make it more efficient. Because we know that this kind of tracing can be very effective, but the GGD does not have the capacity to do this contact-tracing on a larger scale, which would be required once we try to lift the policy measures implementing the intelligent lockdown. However, often very quickly, the discussion goes from automating the work of the GGD, to improving upon it. An app would ‘make us independent of the unreliability of the memory of the patient, and it could trace contacts that the patient wasn’t even aware of’.^10^ This appeal to improvement may be seen as another appeal to the order of industry: making the app more efficient and accurate. However, for health purposes, it is quite uncertain that these implications will improve contact-tracing.^11^ Often, the improvement is framed for the sake of innovation itself, in which case it is an appeal to the ‘project’ order, where innovation and experimentation are in themselves seen as the ultimate goal. Another instance where the project order seems to be mobilized is when it is emphasized that all over the world countries are experimenting, developing and improving these apps, implying that if the Netherlands will not do this, they will be falling behind on innovation.^12^

Finally, a justification analysis of this debate makes clear that the concept of privacy itself is used by mobilizing different orders of worth. As mentioned above, most of the time, concerns for privacy appeal to the civic repertoire. However, at other times privacy is just accepted as an essential part of the Dutch national identity: ‘We have to do this [develop the apps] with one strong and important condition: privacy. Obviously, absolutely. Because that is how we do things here and that is how we want to do this’.^13^ In this instance, where privacy is discussed as a Dutch tradition, it is being discussed by appealing to the domestic repertoire. The domestic repertoire values hierarchy, familial relations (like the state as a father-figure protecting society) as well as tradition and locality. This last element means that some things are valued because they traditionally belong to a group or region. Analyzing the debate with the framework of Boltanski and Thévenot shows us how a concept like privacy is used in these two very distinct ways, one arguing for privacy as a civil right, another arguing for privacy as a part of a cultural heritage. The framework does not offer any tools for evaluating which conception of privacy should be used. Any normative evaluation would be part of a next and separate step.

## A roadmap for the corona-app

Next steps for this research project entail a more detailed justification analysis of the Dutch public debate on the use of contact-tracing apps, which may reveal more conceptions of the common good, and which will clarify which orders of worth are mobilized. The analysis will mainly focus on how government officials argue for the importance of a corona-app in press-briefings for the public, debates in parliament and in the broader public discussion, gleaned from popular media, as well as on the responses by scholars, NGOs and others in the same public debate. This will include opinion pieces published in established newspapers or online, public statements and urgent appeals made to the government. The identification of which common goods are at stake will contribute to discussions about the use of this technology from a standpoint with a richer ethical perspective. Such a perspective is needed for two reasons. First, because it is inclusive to a plurality of ethical concerns. Second, because by offering a map of the common goods at stake, it allows for a more practical starting point from where to think about implementation and design of such an app, where we can consider whether and how to integrate these values. By the same token, this analysis can inform policy on the application of this technology.

## Footnotes

1 Davidson 2020; Hao 2020; Timberg and Harwell 2020; Wetsman 2020).

2 For a nice overview of this and other aspects of the different approaches governments take on this pandemic, see: https://www.cambridge.org/core/blog/tag/country-responses-to-the-covid19-pandemic/.

3 Just a few examples from the dutch debate: NRC, ‘Dit zijn de smaken van ‘epidemiologische surveillance’ waar Nederland uit kan kiezen’ 10–04-2020; BNR, ‘Corona-Data: Gezondheid versus Privacy’ 07–04-2020; Parool, ' In de strijd tegen corona staat privacy op een zijspoor’ 26–03-2020.

4 Trouw, ‘corona vergt tijdelijk een privacy-offer’ 10–04-2020.

5 The dutch phrase usually used is ‘ten koste gaan van’, implying we pay for our health with privacy, or the other way around.

6 The idea that privacy can be seen as a social good was argued for in this debate by M. Lanzing: Kliklijn: een ethisch spoorboekje voor de corona-app in drie scenarios – Bij Nader Inzien (n.d.). Available at: https://bijnaderinzien.com/2020/05/12/kliklijn-een-ethisch-spoorboekje-voor-de-corona-app-in-drie-scenarios/ (accessed 29 May 2020).

7 In later works, the ‘project’ (valuing innovation) and ‘ecological’ orders were later additions to the initial six presented in *On Justification*; (Lafaye and Thévenot, 2017) & (Boltanski and Chiapello, 2005).

8 Original dutch: ‘Het beter kunnen opsporen van besmettingen kan helpen om een nieuwe opleving van de uitbraak te voorkomen’, said during press conference on april 7th, 2020: https://www.youtube.com/watch?v=-j3_mmZcBZU.

9 This line of arguing is present in the press conferences of the government during times of corona, these examples are taken from the press briefing of April 7^th^, 2020: https://www.youtube.com/watch?v=-j3_mmZcBZU.

10 This line of argument can be found in the briefing of parliament on corona-apps: https://www.youtube.com/watch?v=eX0sWkbyKfU.

11 Many people are concerned abouth the efficacy of the apps, an example of such concern is the report by the Ada Lovelace Institute: https://www.adalovelaceinstitute.org/our-work/covid-19/covid-19-exit-through-the-app-store/ (accessed 8 june 2020).

12 These two and other instances of appeals to the project order can be found in the briefing of parliament on corona-apps: https://www.youtube.com/watch?v=eX0sWkbyKfU: ‘We zijn ook niet de enige in europa. Op dit moment hebben Tsjechië, Cyprus, Oostenrijk, Ijsland en Noorwegen al apps geïntroduceerd voor het ondersteunen van het werk van hun ggd. Duitsland had aangekondigd dat rond de Pasen te gaan doen, maar die heeft dat uitgesteld met enkele weken zeggen ze, kortom in heel europa en eigenlijk de hele wereld is men op zoek naar de mogelijkheden om het werk van de ggd, waarvan u net de heer Gouw hoorde zeggen dat het heel intensief is, omddat werk met digitale middelen te ondersteunen.’ This may also be a place where one should consider not orders of worth, but other ‘regimes of engagement’, of which the justification-regime is just one. These arguments used in this discussion seem to follow the grammar of the regime of exploration. For a nice explanation of how the regimes relate to each other, see: Gabowitsch, M. (2020) Regimes of Engagement and Protest in Russia: A Reply to Arnold, Sidorkina, and Shevchenko. *Nationalities Papers* 48(2). Cambridge University Press: 414–418. https://doi.org/10.1017/nps.2019.108.

13 Loosely translated, original Dutch: ‘En we moeten dat doen met als hele harde belangrijke randvoorwaarde: privacy, natuurlijk, absoluut, want want ja zo werkt het nu eenmaal bij ons en zo willen we dat ook laten werken’, citation from minister of health during press briefing of April 7^th^, 2020: https://www.youtube.com/watch?v=-j3_mmZcBZU.
